# 4-(9-Anthr­yl)-1-(2-methoxy­phen­yl)spiro­[azetidin-3,9′-xanthen]-2-one

**DOI:** 10.1107/S1600536809005200

**Published:** 2009-02-28

**Authors:** Şerife Pınar Yalçın, Mehmet Akkurt, Aliasghar Jarrahpour, Edris Ebrahimi, Orhan Büyükgüngör

**Affiliations:** a Department of Physics, Faculty of Arts and Sciences, Erciyes University, 38039 Kayseri, Turkey; b Department of Chemistry, College of Sciences, Shiraz University, 71454 Shiraz, Iran; c Department of Physics, Faculty of Arts and Sciences, Ondokuz Mayıs University, 55139 Samsun, Turkey

## Abstract

The stabilized conformation of the title compound, C_36_H_25_NO_3_, 4-(9-anthryl)-1-(2-methoxyphenyl)-spiro[azetid­in-3,9′-xanthen]-2-one, may be compared with that of the isomeric compound 4-(9-anthr­yl)-1-(4-methoxy­phen­yl)spiro­[azetidin-3,9′-xanthen]-2-one. In the title isomer, the meth­oxy group is slightly twisted out of the plane of the attached benzene ring, with a C—O—C—C torsion angle of 31.5 (2)°. Its β-lactam ring is essentially planar, with a maximum deviation of −0.021 (1) Å. The β-lactam ring makes dihedral angles of 18.815 (9), 83.33 (7) and 53.62 (8)° with the mean planes of the benzene, xanthene and anthracene ring systems, respectively. The structure is stabilized by C—H⋯π, C—H⋯N and C—H⋯O inter­actions.

## Related literature

For general background to β-lactam anti­biotics, see: Banik *et al.* (2004 ▶); Georg & Ravikumar (1993 ▶); Jarrahpour & Khalili (2007 ▶); Palomo *et al.* (2001 ▶). For the crystal structure of the isomeric compound 4-(9-anthr­yl)-1-(4-methoxy­phen­yl)spiro­[azetidin-3,9′-xanthen]-2-one, see: Akkurt, Karaca *et al.* (2008 ▶). For the crystal structures of related compounds, see: Pınar *et al.* (2006 ▶); Akkurt, Jarrahpour *et al.* (2008 ▶). For ring-puckering analysis, see: Cremer & Pople (1975 ▶).
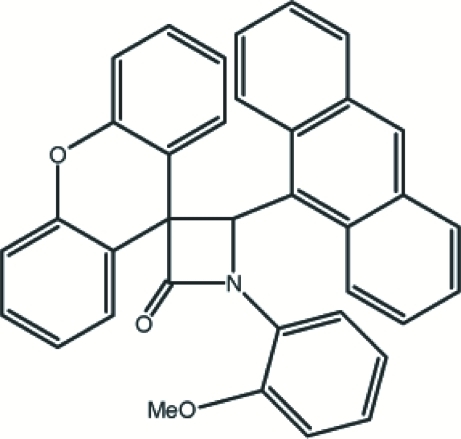


## Experimental

### 

#### Crystal data

C_36_H_25_NO_3_
*M* *_r_* = 519.57
Monoclinic, 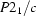

*a* = 11.8496 (3) Å
*b* = 15.3168 (5) Å
*c* = 14.9883 (4) Å
β = 106.536 (2)°
*V* = 2607.84 (13) Å^3^
*Z* = 4
Mo *K*α radiation
μ = 0.08 mm^−1^
*T* = 295 K
0.62 × 0.59 × 0.56 mm

#### Data collection

Stoe IPDS-II diffractometer
Absorption correction: integration (*X-RED32*; Stoe & Cie, 2002 ▶) *T*_min_ = 0.950, *T*_max_ = 0.955
32362 measured reflections
5399 independent reflections
4314 reflections with *I* > 2σ(*I*)
*R*_int_ = 0.054

#### Refinement

*R*[*F* ^2^ > 2σ(*F* ^2^)] = 0.041
*wR*(*F* ^2^) = 0.106
*S* = 1.04
5399 reflections
363 parameters
H-atom parameters constrained
Δρ_max_ = 0.16 e Å^−3^
Δρ_min_ = −0.16 e Å^−3^

### 

Data collection: *X-AREA* (Stoe & Cie, 2002 ▶); cell refinement: *X-AREA*; data reduction: *X-RED32* (Stoe & Cie, 2002 ▶); program(s) used to solve structure: *SIR97* (Altomare *et al.*, 1999 ▶); program(s) used to refine structure: *SHELXL97* (Sheldrick, 2008 ▶); molecular graphics: *ORTEP-3* (Farrugia, 1997 ▶); software used to prepare material for publication: *WinGX* (Farrugia, 1999 ▶).

## Supplementary Material

Crystal structure: contains datablocks global, I. DOI: 10.1107/S1600536809005200/sj2572sup1.cif

Structure factors: contains datablocks I. DOI: 10.1107/S1600536809005200/sj2572Isup2.hkl

Additional supplementary materials:  crystallographic information; 3D view; checkCIF report

## Figures and Tables

**Table 1:** Hydrogen-bond geometry (Å, °)

*D*—H⋯*A*	*D*—H	H⋯*A*	*D*⋯*A*	*D*—H⋯*A*
C2—H2⋯N1	0.93	2.32	2.9236 (19)	122
C31—H31⋯O2	0.93	2.55	3.140 (2)	122
C5—H5⋯*Cg*1^i^	0.93	2.92	3.597 (2)	130
C26—H26⋯*Cg*2^ii^	0.93	2.88	3.7180 (19)	151

Symmetry codes: (i) 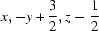; (ii) 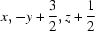. *Cg*1 and *Cg*2 are the centroids of the C30–C35 and C8–C13 rings, respectively.

**Fig. 1.**
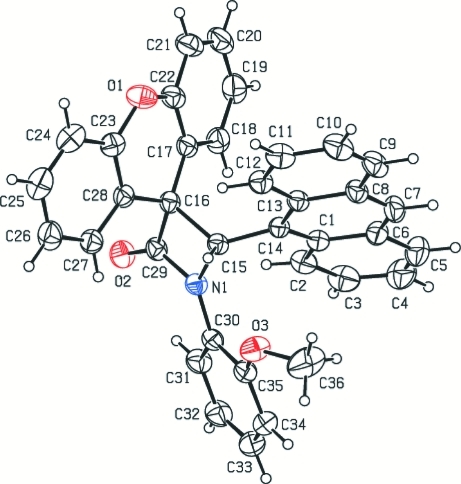
The title molecular structure, with the atom-numbering scheme and 30% probability displacement ellipsoids

**Fig. 2.**
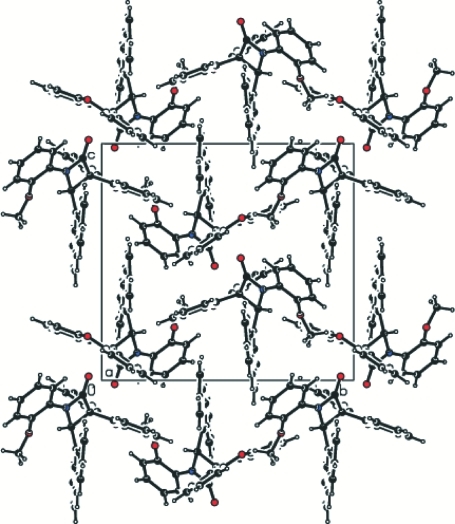
A view down the a axis of the packing of (I).

## supplementary crystallographic information

### Comment

The importance of β-lactams for the treatment of bacterial infections has been amply established (Georg & Ravikumar, 1993; Palomo *et al.*, 2001). Large efforts have been made for the synthesis and structural modification of the β-lactam nucleus to increase antimicrobial activity. New and interesting spiro-β-lactam-containing structures have been recently reported due to their structural features and their bioactivity (Jarrahpour & Khalili, 2007; Pınar *et al.*, 2006; Akkurt, Jarrahpour *et al.*, 2008; Akkurt, Karaca *et al.*, 2008). Banik and coworkers have synthesized polycyclic aroaromatic β-lactams with potential anticancer properties (Banik *et al.*, 2004). The title compound (I) is isomeric with 4-(9-anthryl)-1-(4-methoxyphenyl)spiro[azetidin-3,9'-xanthen]-2-one (II), whose crystal structure has been previously reported by Akkurt, Karaca *et al.* (2008). Both isomers crystalize in the monoclinic space group *P*2_1_/*c*. The volume of the unit cell of the title isomer (I) is smaller than that of (II).

As can be seen in Fig. 1, the β-lactam ring of (I) is nearly planar, with a maximum deviation of -0.021 (1) Å from the ring. The planarity is mainly due to the *sp*^2^ hybridization of atoms C29 and N1. The dihedral angle between the benzene ring C30—C35 attached at N1 and the β-lactam ring is 58.80 (6)°, in the isomeric compound (II), the equivalent angle is 28.45 (14)° (Akkurt, Karaca *et al.*, 2008) due to steric interactions resulting in different orientations of the methoxy group. The methoxy group is slightly twisted out of the plane of the attached benzene ring [C36—O3—C35—C34 = 31.5 (2) °, and in (II) -4.6 (4)].

In the xanthene ring system, attached at C16, the benzene rings (C17–C22) and (C23–C28) form a dihedral angle of 16.78 (8)° with each other. The central ring, C16/C17/C22/O1/C23/C28, is not planar, with puckering parameters: Q_T_ = 0.2173 (14) Å, θ = 95.6 (4)° and φ = 358.3 (4)° (Cremer & Pople, 1975). The mean plane of the xanthene ring system forms dihedral angles of 83.33 (7)°, and 65.92 (6)°, with the β-lactam ring and the benzene ring C30–C35, respectively.

The anthracene ring system, attached at C15, is almost planar, with maximum deviations of -0.074 (2) Å for C2, 0.046 (2) Å for C4, 0.040 (2) Å for C5 and -0.055 (1) Å for C8. It forms dihedral angles of 53.62 (8)°, 58.80 (6)° and 69.89 (4)°, with the β-lactam ring, benzene ring (C30–C35) and the mean plane of the xanthene ring system, respectively.

The molecular structure of the title compound is stabilized by two intramolecular C—H···O and C—H···N hydrogen bonding interactions (Table 1). Fig. 2 shows the packing diagram viewed down the *a* axis. Two C—H···π interactions are also found between C5–H5 and the centroid of the C30–C35 benzene ring, and C26–H26 and the centroid of the C8–C13 benzene ring.

### Experimental

A mixture of (*E*)—*N*-(antheracen-9-ylmethylene)-2-methoxyaniline (0.30 g, 0.96 mmol) and triethylamine (0.73 g,7.21 mmol), 9*H*-xanthen-9-carboxylic acid (0.49 g, 2.17 mmol) and tosylchloride (0.42 g, 2.20 mmol) in CH_2_Cl_2_(15 ml) was strirred at room temperature for 24 h. It was then washed with 1M HCl (20 ml) and saturated sodium bicarbonate solution (20 ml), brine (20 ml), dried (Na_2_SO_4_) and the solvent was evaporated to give the crude product as light yellow crystals which were then purified by recrystallization from ethyl acetate (Yield 57%). dec.: 507–509 K. IR (KBr, cm^-1^):1739 (CO β-lactam). ^1^H-NMR δ (p.p.m.): 2.94 (s, 3H, OCH_3_), 6.34 (s, 1H,4), 6.55–9.20 (m, ArH, 21H).^13^C-NMR δ (p.p.m.): 55.4 (OCH_3_),66.0 (C-3), 78.5 (C-4), 112.8–152.0 (aromatic carbon), 168.0 (CO β-lactam). Analysis calculated for C_36_H_25_NO_3_: C 83.22, H 4.85, N 2.70%. Found: C 83.90, H 4.80, N 2.81%.

### Refinement

The H atoms were positioned geometrically and refined using a riding model, with C—H = 0.93–0.98 Å and *U*_iso_(H) = 1.2*U*_eq_(C).

### Crystal data

**Table:**

C_36_H_25_NO_3_	*F*(000) = 1088
*M**_r_* = 519.57	*D*_x_ = 1.323 Mg m^−^^3^
Monoclinic, *P*2_1_/*c*	Mo *K*α radiation, λ = 0.71073 Å
Hall symbol: -P 2ybc	Cell parameters from 32362 reflections
*a* = 11.8496 (3) Å	θ = 1.4–28.0°
*b* = 15.3168 (5) Å	µ = 0.08 mm^−^^1^
*c* = 14.9883 (4) Å	*T* = 295 K
β = 106.536 (2)°	Prism, colourless
*V* = 2607.84 (13) Å^3^	0.62 × 0.59 × 0.56 mm
*Z* = 4	

### Data collection

**Table:**

STOE IPDS-II diffractometer	5399 independent reflections
Radiation source: sealed X-ray tube, 12 x 0.4 mm long-fine focus	4314 reflections with *I* > 2σ(*I*)
plane graphite	*R*_int_ = 0.054
Detector resolution: 6.67 pixels mm^-1^	θ_max_ = 26.5°, θ_min_ = 1.8°
ω scans	*h* = −14→14
Absorption correction: integration (*X-RED32*; Stoe & Cie, 2002)	*k* = −19→19
*T*_min_ = 0.950, *T*_max_ = 0.955	*l* = −18→18
32362 measured reflections	

### Refinement

**Table:**

Refinement on *F*^2^	Secondary atom site location: difference Fourier map
Least-squares matrix: full	Hydrogen site location: inferred from neighbouring sites
*R*[*F*^2^ > 2σ(*F*^2^)] = 0.041	H-atom parameters constrained
*wR*(*F*^2^) = 0.106	*w* = 1/[σ^2^(*F*_o_^2^) + (0.048*P*)^2^ + 0.3336*P*] where *P* = (*F*_o_^2^ + 2*F*_c_^2^)/3
*S* = 1.04	(Δ/σ)_max_ < 0.001
5399 reflections	Δρ_max_ = 0.16 e Å^−^^3^
363 parameters	Δρ_min_ = −0.16 e Å^−^^3^
0 restraints	Extinction correction: *SHELXL*, FC^*^=KFC[1+0.001XFC^2^Λ^3^/SIN(2Θ)]^-1/4^
Primary atom site location: structure-invariant direct methods	Extinction coefficient: 0.0105 (9)

### Special details

**Table:**

Geometry. Bond distances, angles *etc*. have been calculated using the rounded fractional coordinates. All su's are estimated from the variances of the (full) variance-covariance matrix. The cell e.s.d.'s are taken into account in the estimation of distances, angles and torsion angles
Refinement. Refinement on *F*^2^ for ALL reflections except those flagged by the user for potential systematic errors. Weighted *R*-factors *wR* and all goodnesses of fit *S* are based on *F*^2^, conventional *R*-factors *R* are based on *F*, with *F* set to zero for negative *F*^2^. The observed criterion of *F*^2^ > σ(*F*^2^) is used only for calculating -*R*-factor-obs *etc*. and is not relevant to the choice of reflections for refinement. *R*-factors based on *F*^2^ are statistically about twice as large as those based on *F*, and *R*-factors based on ALL data will be even larger.

### Fractional atomic coordinates and isotropic or equivalent isotropic displacement parameters (Å^2^)

**Table:**

	*x*	*y*	*z*	*U*_iso_*/*U*_eq_	
O1	−0.01289 (9)	0.44378 (8)	0.31404 (8)	0.0697 (4)	
O2	0.36695 (10)	0.55177 (7)	0.51655 (7)	0.0653 (4)	
O3	0.30250 (9)	0.78834 (7)	0.27655 (8)	0.0675 (4)	
N1	0.33576 (9)	0.64088 (7)	0.38357 (7)	0.0461 (3)	
C1	0.36306 (11)	0.58740 (9)	0.19255 (9)	0.0462 (4)	
C2	0.47171 (12)	0.57344 (10)	0.26356 (11)	0.0569 (5)	
C3	0.57582 (14)	0.56915 (12)	0.24285 (15)	0.0751 (7)	
C4	0.58118 (17)	0.57587 (14)	0.15052 (17)	0.0884 (8)	
C5	0.48186 (17)	0.58388 (13)	0.08111 (15)	0.0792 (7)	
C6	0.36987 (14)	0.58871 (10)	0.09806 (11)	0.0568 (5)	
C7	0.26796 (15)	0.59596 (11)	0.02468 (10)	0.0626 (5)	
C8	0.15810 (13)	0.60135 (9)	0.03888 (9)	0.0531 (4)	
C9	0.05274 (17)	0.60473 (11)	−0.03720 (10)	0.0675 (6)	
C10	−0.05367 (16)	0.60989 (11)	−0.02326 (11)	0.0705 (6)	
C11	−0.06362 (14)	0.61307 (11)	0.06781 (12)	0.0647 (5)	
C12	0.03379 (12)	0.61083 (9)	0.14315 (10)	0.0520 (4)	
C13	0.14958 (11)	0.60449 (8)	0.13233 (8)	0.0446 (4)	
C14	0.25263 (11)	0.60084 (8)	0.20881 (8)	0.0415 (3)	
C15	0.23391 (11)	0.62055 (8)	0.30239 (8)	0.0418 (4)	
C16	0.20143 (11)	0.54586 (8)	0.36502 (8)	0.0438 (4)	
C17	0.19653 (12)	0.45304 (9)	0.33143 (9)	0.0481 (4)	
C18	0.29543 (15)	0.40863 (10)	0.32265 (11)	0.0604 (5)	
C19	0.28781 (19)	0.32418 (11)	0.28933 (12)	0.0751 (7)	
C20	0.1806 (2)	0.28190 (11)	0.26398 (12)	0.0779 (7)	
C21	0.08265 (17)	0.32332 (11)	0.27326 (11)	0.0706 (6)	
C22	0.09090 (13)	0.40801 (10)	0.30662 (9)	0.0554 (5)	
C23	−0.00679 (12)	0.51767 (10)	0.36724 (10)	0.0543 (5)	
C24	−0.10730 (14)	0.53833 (12)	0.39252 (13)	0.0696 (6)	
C25	−0.10924 (16)	0.61064 (12)	0.44524 (14)	0.0754 (7)	
C26	−0.01121 (17)	0.66294 (12)	0.47321 (13)	0.0725 (6)	
C27	0.08836 (14)	0.64221 (10)	0.44783 (11)	0.0588 (5)	
C28	0.09329 (12)	0.56874 (9)	0.39433 (9)	0.0475 (4)	
C29	0.31556 (12)	0.57514 (9)	0.43826 (9)	0.0478 (4)	
C30	0.42510 (11)	0.70525 (9)	0.39776 (9)	0.0469 (4)	
C31	0.53095 (13)	0.69324 (11)	0.46667 (10)	0.0595 (5)	
C32	0.62199 (13)	0.75191 (12)	0.47606 (12)	0.0676 (6)	
C33	0.60857 (14)	0.82195 (13)	0.41712 (12)	0.0692 (6)	
C34	0.50341 (14)	0.83551 (11)	0.34932 (11)	0.0625 (5)	
C35	0.41095 (12)	0.77789 (9)	0.33996 (9)	0.0509 (4)	
C36	0.2984 (2)	0.82970 (17)	0.19185 (16)	0.1126 (9)	
H2	0.47080	0.56720	0.32510	0.0680*	
H3	0.64500	0.56160	0.29060	0.0900*	
H4	0.65360	0.57470	0.13780	0.1060*	
H5	0.48610	0.58640	0.02010	0.0950*	
H7	0.27380	0.59720	−0.03590	0.0750*	
H9	0.05840	0.60330	−0.09780	0.0810*	
H10	−0.12090	0.61140	−0.07380	0.0850*	
H11	−0.13780	0.61680	0.07700	0.0780*	
H12	0.02460	0.61350	0.20270	0.0620*	
H15	0.17610	0.66770	0.29470	0.0500*	
H18	0.36820	0.43650	0.33960	0.0720*	
H19	0.35490	0.29580	0.28400	0.0900*	
H20	0.17520	0.22530	0.24060	0.0930*	
H21	0.01050	0.29470	0.25720	0.0850*	
H24	−0.17350	0.50290	0.37350	0.0840*	
H25	−0.17670	0.62450	0.46220	0.0900*	
H26	−0.01220	0.71220	0.50920	0.0870*	
H27	0.15410	0.67820	0.46690	0.0700*	
H31	0.54040	0.64550	0.50650	0.0710*	
H32	0.69250	0.74390	0.52240	0.0810*	
H33	0.67070	0.86060	0.42280	0.0830*	
H34	0.49480	0.88350	0.30990	0.0750*	
H36A	0.35790	0.80540	0.16710	0.1350*	
H36B	0.31220	0.89110	0.20230	0.1350*	
H36C	0.22230	0.82080	0.14840	0.1350*	

### Atomic displacement parameters (Å^2^)

**Table:**

	*U*^11^	*U*^22^	*U*^33^	*U*^12^	*U*^13^	*U*^23^
O1	0.0557 (6)	0.0655 (7)	0.0769 (8)	−0.0070 (5)	0.0011 (5)	−0.0118 (6)
O2	0.0716 (7)	0.0750 (7)	0.0390 (5)	−0.0004 (5)	−0.0009 (5)	0.0127 (5)
O3	0.0614 (6)	0.0611 (6)	0.0668 (7)	−0.0036 (5)	−0.0031 (5)	0.0181 (5)
N1	0.0486 (6)	0.0482 (6)	0.0351 (5)	−0.0030 (5)	0.0014 (4)	0.0024 (5)
C1	0.0472 (7)	0.0424 (7)	0.0483 (7)	−0.0006 (5)	0.0123 (6)	−0.0015 (6)
C2	0.0488 (8)	0.0543 (8)	0.0643 (9)	0.0050 (6)	0.0106 (7)	−0.0072 (7)
C3	0.0470 (8)	0.0730 (11)	0.1011 (14)	0.0045 (7)	0.0142 (9)	−0.0145 (10)
C4	0.0643 (11)	0.0941 (14)	0.1214 (18)	−0.0015 (10)	0.0499 (12)	−0.0093 (13)
C5	0.0783 (12)	0.0893 (13)	0.0844 (13)	−0.0026 (10)	0.0464 (10)	−0.0024 (10)
C6	0.0645 (9)	0.0543 (8)	0.0581 (8)	−0.0024 (7)	0.0280 (7)	0.0001 (7)
C7	0.0828 (11)	0.0667 (10)	0.0409 (7)	0.0005 (8)	0.0217 (7)	0.0020 (7)
C8	0.0677 (9)	0.0498 (7)	0.0371 (7)	0.0005 (6)	0.0073 (6)	0.0024 (6)
C9	0.0905 (12)	0.0628 (9)	0.0369 (7)	0.0051 (8)	−0.0019 (7)	0.0024 (7)
C10	0.0688 (11)	0.0703 (10)	0.0527 (9)	0.0068 (8)	−0.0147 (8)	0.0012 (8)
C11	0.0511 (8)	0.0640 (10)	0.0661 (10)	0.0047 (7)	−0.0039 (7)	0.0028 (8)
C12	0.0486 (7)	0.0551 (8)	0.0467 (7)	0.0010 (6)	0.0044 (6)	0.0021 (6)
C13	0.0502 (7)	0.0416 (6)	0.0372 (6)	0.0000 (5)	0.0047 (5)	0.0018 (5)
C14	0.0448 (6)	0.0403 (6)	0.0363 (6)	0.0001 (5)	0.0068 (5)	0.0029 (5)
C15	0.0424 (6)	0.0437 (7)	0.0350 (6)	0.0007 (5)	0.0039 (5)	0.0011 (5)
C16	0.0488 (7)	0.0452 (7)	0.0349 (6)	0.0010 (5)	0.0080 (5)	0.0035 (5)
C17	0.0617 (8)	0.0447 (7)	0.0355 (6)	0.0024 (6)	0.0101 (6)	0.0052 (5)
C18	0.0751 (10)	0.0520 (8)	0.0581 (9)	0.0089 (7)	0.0254 (7)	0.0086 (7)
C19	0.1100 (14)	0.0555 (9)	0.0693 (11)	0.0218 (10)	0.0410 (10)	0.0092 (8)
C20	0.1310 (17)	0.0479 (9)	0.0537 (9)	0.0050 (10)	0.0247 (10)	−0.0042 (7)
C21	0.0950 (12)	0.0539 (9)	0.0527 (9)	−0.0102 (9)	0.0044 (8)	−0.0038 (7)
C22	0.0668 (9)	0.0511 (8)	0.0414 (7)	−0.0004 (7)	0.0042 (6)	0.0011 (6)
C23	0.0528 (8)	0.0527 (8)	0.0530 (8)	0.0018 (6)	0.0078 (6)	0.0082 (7)
C24	0.0490 (8)	0.0744 (11)	0.0828 (11)	0.0058 (7)	0.0144 (8)	0.0221 (9)
C25	0.0716 (11)	0.0748 (12)	0.0890 (13)	0.0240 (9)	0.0378 (10)	0.0247 (10)
C26	0.0927 (12)	0.0606 (10)	0.0748 (11)	0.0173 (9)	0.0411 (10)	0.0069 (8)
C27	0.0733 (9)	0.0505 (8)	0.0576 (9)	0.0016 (7)	0.0269 (7)	0.0007 (7)
C28	0.0545 (8)	0.0471 (7)	0.0412 (7)	0.0013 (6)	0.0141 (6)	0.0063 (6)
C29	0.0527 (7)	0.0509 (7)	0.0366 (6)	0.0032 (6)	0.0077 (5)	0.0025 (6)
C30	0.0470 (7)	0.0505 (7)	0.0401 (7)	−0.0019 (6)	0.0073 (5)	−0.0051 (6)
C31	0.0560 (8)	0.0654 (9)	0.0476 (8)	−0.0018 (7)	−0.0004 (6)	−0.0011 (7)
C32	0.0479 (8)	0.0869 (12)	0.0600 (9)	−0.0063 (8)	0.0025 (7)	−0.0119 (9)
C33	0.0535 (8)	0.0846 (12)	0.0700 (10)	−0.0185 (8)	0.0185 (7)	−0.0104 (9)
C34	0.0654 (9)	0.0606 (9)	0.0635 (9)	−0.0097 (7)	0.0218 (8)	0.0011 (7)
C35	0.0507 (7)	0.0529 (8)	0.0466 (7)	−0.0011 (6)	0.0100 (6)	−0.0024 (6)
C36	0.1148 (17)	0.1056 (17)	0.0883 (15)	−0.0211 (13)	−0.0180 (13)	0.0514 (13)

### Geometric parameters (Å, °)

**Table:**

O1—C22	1.3795 (19)	C23—C28	1.382 (2)
O1—C23	1.3745 (19)	C24—C25	1.365 (3)
O2—C29	1.2121 (17)	C25—C26	1.375 (3)
O3—C35	1.3730 (18)	C26—C27	1.376 (3)
O3—C36	1.407 (3)	C27—C28	1.393 (2)
N1—C15	1.4826 (16)	C30—C31	1.392 (2)
N1—C29	1.3619 (17)	C30—C35	1.3906 (19)
N1—C30	1.4177 (17)	C31—C32	1.380 (2)
C1—C2	1.434 (2)	C32—C33	1.370 (3)
C1—C6	1.441 (2)	C33—C34	1.381 (2)
C1—C14	1.4127 (19)	C34—C35	1.383 (2)
C2—C3	1.357 (2)	C2—H2	0.9300
C3—C4	1.407 (3)	C3—H3	0.9300
C4—C5	1.336 (3)	C4—H4	0.9300
C5—C6	1.422 (3)	C5—H5	0.9300
C6—C7	1.387 (2)	C7—H7	0.9300
C7—C8	1.380 (2)	C9—H9	0.9300
C8—C9	1.432 (2)	C10—H10	0.9300
C8—C13	1.4335 (18)	C11—H11	0.9300
C9—C10	1.338 (3)	C12—H12	0.9300
C10—C11	1.404 (2)	C15—H15	0.9800
C11—C12	1.366 (2)	C18—H18	0.9300
C12—C13	1.430 (2)	C19—H19	0.9300
C13—C14	1.4183 (17)	C20—H20	0.9300
C14—C15	1.5115 (17)	C21—H21	0.9300
C15—C16	1.5949 (17)	C24—H24	0.9300
C16—C17	1.5038 (18)	C25—H25	0.9300
C16—C28	1.509 (2)	C26—H26	0.9300
C16—C29	1.5460 (19)	C27—H27	0.9300
C17—C18	1.393 (2)	C31—H31	0.9300
C17—C22	1.384 (2)	C32—H32	0.9300
C18—C19	1.380 (2)	C33—H33	0.9300
C19—C20	1.380 (3)	C34—H34	0.9300
C20—C21	1.364 (3)	C36—H36A	0.9600
C21—C22	1.384 (2)	C36—H36B	0.9600
C23—C24	1.385 (2)	C36—H36C	0.9600
			
O2···C31	3.140 (2)	C27···H15	2.8000
O3···C15	2.7559 (16)	C27···H36C^v^	3.0300
O3···N1	2.7331 (15)	C28···H12	2.8400
O3···C14	3.0474 (16)	C29···H27	2.6100
O2···H31	2.5500	C29···H18	2.7600
O3···H15	2.4400	C29···H31	2.7800
N1···O3	2.7331 (15)	C29···H2	2.8400
N1···C2	2.9236 (19)	C30···H2	2.5100
N1···C27	3.336 (2)	C31···H2	2.8100
N1···H2	2.3200	C33···H5^v^	2.7800
N1···H27	2.8400	C34···H36A	2.8200
C1···C18	3.582 (2)	C34···H36B	2.8100
C1···C30	3.4600 (19)	C34···H19^ix^	3.0200
C2···N1	2.9236 (19)	C34···H5^v^	2.8900
C2···C18	3.546 (2)	C36···H34	2.6200
C2···C30	3.010 (2)	C36···H24^x^	3.0600
C2···C31	3.451 (2)	H2···N1	2.3200
C2···C35	3.479 (2)	H2···C15	2.8500
C5···C33^i^	3.532 (3)	H2···C29	2.8400
C8···C10^ii^	3.448 (2)	H2···C30	2.5100
C9···C11^ii^	3.375 (2)	H2···C31	2.8100
C9···C10^ii^	3.409 (2)	H2···H18	2.3800
C10···C8^ii^	3.448 (2)	H3···C24^xi^	2.9200
C10···C9^ii^	3.409 (2)	H3···H24^xi^	2.3400
C10···C10^ii^	3.596 (2)	H5···H7	2.4200
C10···C26^i^	3.518 (3)	H5···C5^vii^	3.0900
C11···C9^ii^	3.375 (2)	H5···C33^i^	2.7800
C12···C16	3.4978 (19)	H5···C34^i^	2.8900
C14···O3	3.0474 (16)	H7···H5	2.4200
C14···C36	3.567 (3)	H7···H9	2.4600
C14···C35	3.5551 (18)	H9···H7	2.4600
C14···C18	3.368 (2)	H9···C21^ii^	2.9100
C15···O3	2.7559 (16)	H10···H33^xii^	2.4900
C16···C12	3.4978 (19)	H12···C15	2.5100
C18···C14	3.368 (2)	H12···C16	2.9100
C18···C31^iii^	3.597 (2)	H12···C23	2.9800
C18···C2	3.546 (2)	H12···C28	2.8400
C18···C1	3.582 (2)	H12···H15	2.1000
C19···C32^iii^	3.567 (2)	H15···O3	2.4400
C23···C24^iv^	3.572 (2)	H15···C12	2.5700
C23···C25^iv^	3.384 (2)	H15···C27	2.8000
C24···C23^iv^	3.572 (2)	H15···H12	2.1000
C24···C28^iv^	3.553 (2)	H18···C2	2.8300
C25···C23^iv^	3.384 (2)	H18···C29	2.7600
C26···C10^v^	3.518 (3)	H18···H2	2.3800
C27···N1	3.336 (2)	H18···H31^iii^	2.5800
C28···C24^iv^	3.553 (2)	H19···C34^vi^	3.0200
C30···C2	3.010 (2)	H24···H3^viii^	2.3400
C30···C1	3.4600 (19)	H24···C36^xiii^	3.0600
C31···C2	3.451 (2)	H24···H36B^xiii^	2.4200
C31···C18^iii^	3.597 (2)	H26···C9^v^	3.0400
C31···O2	3.140 (2)	H26···C10^v^	2.7900
C32···C19^iii^	3.567 (2)	H26···C11^v^	2.9300
C33···C5^v^	3.532 (3)	H27···N1	2.8400
C35···C14	3.5551 (18)	H27···C15	3.0100
C35···C2	3.479 (2)	H27···C29	2.6100
C36···C14	3.567 (3)	H31···O2	2.5500
C2···H18	2.8300	H31···C29	2.7800
C3···H34^vi^	3.0100	H31···C18^iii^	2.8600
C3···H36B^vi^	3.0400	H31···H18^iii^	2.5800
C5···H5^vii^	3.0900	H32···C19^iii^	2.9500
C9···H26^i^	3.0400	H33···H10^xiv^	2.4900
C10···H26^i^	2.7900	H34···C36	2.6200
C11···H26^i^	2.9300	H34···H36A	2.5800
C12···H15	2.5700	H34···H36B	2.3000
C15···H27	3.0100	H34···C3^ix^	3.0100
C15···H2	2.8500	H36A···C34	2.8200
C15···H12	2.5100	H36A···H34	2.5800
C16···H12	2.9100	H36B···C34	2.8100
C18···H31^iii^	2.8600	H36B···H34	2.3000
C19···H32^iii^	2.9500	H36B···C3^ix^	3.0400
C21···H9^ii^	2.9100	H36B···H24^x^	2.4200
C23···H12	2.9800	H36C···C27^i^	3.0300
C24···H3^viii^	2.9200		
			
C22—O1—C23	118.22 (12)	N1—C30—C31	119.75 (12)
C35—O3—C36	117.53 (14)	N1—C30—C35	120.70 (12)
C15—N1—C29	95.58 (10)	C31—C30—C35	119.44 (13)
C15—N1—C30	131.90 (10)	C30—C31—C32	120.19 (15)
C29—N1—C30	132.50 (11)	C31—C32—C33	120.02 (16)
C2—C1—C6	116.22 (13)	C32—C33—C34	120.47 (17)
C2—C1—C14	125.01 (12)	C33—C34—C35	120.11 (15)
C6—C1—C14	118.76 (12)	O3—C35—C30	116.59 (12)
C1—C2—C3	121.35 (15)	O3—C35—C34	123.67 (13)
C2—C3—C4	121.33 (18)	C30—C35—C34	119.73 (13)
C3—C4—C5	119.8 (2)	C1—C2—H2	119.00
C4—C5—C6	121.7 (2)	C3—C2—H2	119.00
C1—C6—C5	119.39 (15)	C2—C3—H3	119.00
C1—C6—C7	120.04 (15)	C4—C3—H3	119.00
C5—C6—C7	120.56 (16)	C3—C4—H4	120.00
C6—C7—C8	121.94 (14)	C5—C4—H4	120.00
C7—C8—C9	121.75 (13)	C4—C5—H5	119.00
C7—C8—C13	118.98 (13)	C6—C5—H5	119.00
C9—C8—C13	119.27 (14)	C6—C7—H7	119.00
C8—C9—C10	121.64 (14)	C8—C7—H7	119.00
C9—C10—C11	119.84 (16)	C8—C9—H9	119.00
C10—C11—C12	121.18 (16)	C10—C9—H9	119.00
C11—C12—C13	121.34 (13)	C9—C10—H10	120.00
C8—C13—C12	116.73 (12)	C11—C10—H10	120.00
C8—C13—C14	120.30 (12)	C10—C11—H11	119.00
C12—C13—C14	122.97 (11)	C12—C11—H11	119.00
C1—C14—C13	119.54 (11)	C11—C12—H12	119.00
C1—C14—C15	125.24 (11)	C13—C12—H12	119.00
C13—C14—C15	114.97 (11)	N1—C15—H15	109.00
N1—C15—C14	120.22 (11)	C14—C15—H15	109.00
N1—C15—C16	86.79 (8)	C16—C15—H15	109.00
C14—C15—C16	121.78 (10)	C17—C18—H18	119.00
C15—C16—C17	118.29 (10)	C19—C18—H18	119.00
C15—C16—C28	111.83 (10)	C18—C19—H19	120.00
C15—C16—C29	84.32 (9)	C20—C19—H19	120.00
C17—C16—C28	111.21 (11)	C19—C20—H20	120.00
C17—C16—C29	117.04 (11)	C21—C20—H20	120.00
C28—C16—C29	111.69 (10)	C20—C21—H21	120.00
C16—C17—C18	122.67 (13)	C22—C21—H21	120.00
C16—C17—C22	120.45 (13)	C23—C24—H24	120.00
C18—C17—C22	116.87 (13)	C25—C24—H24	120.00
C17—C18—C19	121.44 (17)	C24—C25—H25	120.00
C18—C19—C20	119.99 (19)	C26—C25—H25	120.00
C19—C20—C21	119.81 (16)	C25—C26—H26	120.00
C20—C21—C22	119.88 (17)	C27—C26—H26	120.00
O1—C22—C17	122.63 (13)	C26—C27—H27	119.00
O1—C22—C21	115.38 (15)	C28—C27—H27	119.00
C17—C22—C21	121.99 (15)	C30—C31—H31	120.00
O1—C23—C24	116.07 (14)	C32—C31—H31	120.00
O1—C23—C28	122.39 (13)	C31—C32—H32	120.00
C24—C23—C28	121.54 (14)	C33—C32—H32	120.00
C23—C24—C25	120.09 (17)	C32—C33—H33	120.00
C24—C25—C26	119.84 (18)	C34—C33—H33	120.00
C25—C26—C27	119.86 (17)	C33—C34—H34	120.00
C26—C27—C28	121.70 (15)	C35—C34—H34	120.00
C16—C28—C23	120.78 (12)	O3—C36—H36A	109.00
C16—C28—C27	122.24 (13)	O3—C36—H36B	109.00
C23—C28—C27	116.97 (14)	O3—C36—H36C	109.00
O2—C29—N1	132.46 (13)	H36A—C36—H36B	109.00
O2—C29—C16	134.32 (13)	H36A—C36—H36C	110.00
N1—C29—C16	93.16 (10)	H36B—C36—H36C	109.00
			
C23—O1—C22—C21	164.09 (13)	N1—C15—C16—C28	108.45 (11)
C22—O1—C23—C24	−163.94 (14)	C14—C15—C16—C17	3.56 (18)
C22—O1—C23—C28	16.0 (2)	N1—C15—C16—C29	−2.66 (9)
C23—O1—C22—C17	−15.4 (2)	C14—C15—C16—C28	−127.61 (12)
C36—O3—C35—C34	31.5 (2)	C14—C15—C16—C29	121.28 (12)
C36—O3—C35—C30	−149.38 (16)	C29—C16—C28—C27	31.69 (17)
C29—N1—C30—C35	−164.50 (14)	C15—C16—C29—O2	−179.64 (17)
C29—N1—C15—C16	3.02 (10)	C15—C16—C28—C23	117.93 (13)
C30—N1—C15—C14	56.11 (18)	C15—C16—C28—C27	−60.86 (16)
C29—N1—C30—C31	19.3 (2)	C28—C16—C17—C22	17.30 (16)
C30—N1—C15—C16	−178.59 (13)	C29—C16—C17—C18	−33.39 (18)
C15—N1—C30—C35	17.7 (2)	C29—C16—C17—C22	147.33 (13)
C15—N1—C29—C16	−3.11 (10)	C15—C16—C17—C18	65.15 (17)
C30—N1—C29—C16	178.51 (13)	C17—C16—C28—C23	−16.74 (17)
C29—N1—C15—C14	−122.28 (12)	C17—C16—C28—C27	164.47 (13)
C15—N1—C29—O2	179.35 (17)	C29—C16—C28—C23	−149.52 (13)
C15—N1—C30—C31	−158.49 (13)	C28—C16—C17—C18	−163.42 (13)
C30—N1—C29—O2	1.0 (3)	C15—C16—C17—C22	−114.14 (14)
C2—C1—C14—C13	−173.20 (13)	C28—C16—C29—N1	−108.37 (11)
C6—C1—C14—C15	−166.21 (12)	C17—C16—C29—O2	−60.7 (2)
C14—C1—C6—C5	173.86 (14)	C17—C16—C29—N1	121.83 (12)
C14—C1—C6—C7	−5.2 (2)	C15—C16—C29—N1	2.89 (10)
C2—C1—C6—C7	175.67 (14)	C28—C16—C29—O2	69.1 (2)
C2—C1—C6—C5	−5.3 (2)	C16—C17—C22—C21	178.37 (13)
C6—C1—C14—C13	7.72 (19)	C18—C17—C22—O1	178.49 (13)
C14—C1—C2—C3	−173.76 (15)	C16—C17—C18—C19	−178.29 (14)
C2—C1—C14—C15	12.9 (2)	C22—C17—C18—C19	1.0 (2)
C6—C1—C2—C3	5.3 (2)	C16—C17—C22—O1	−2.2 (2)
C1—C2—C3—C4	−1.7 (3)	C18—C17—C22—C21	−1.0 (2)
C2—C3—C4—C5	−2.2 (3)	C17—C18—C19—C20	−0.1 (3)
C3—C4—C5—C6	2.1 (3)	C18—C19—C20—C21	−1.0 (3)
C4—C5—C6—C1	1.8 (3)	C19—C20—C21—C22	1.0 (2)
C4—C5—C6—C7	−179.22 (18)	C20—C21—C22—O1	−179.54 (14)
C5—C6—C7—C8	−179.57 (16)	C20—C21—C22—C17	−0.1 (2)
C1—C6—C7—C8	−0.6 (2)	O1—C23—C28—C16	0.9 (2)
C6—C7—C8—C13	3.6 (2)	C24—C23—C28—C16	−179.17 (14)
C6—C7—C8—C9	−177.04 (15)	C24—C23—C28—C27	−0.3 (2)
C7—C8—C13—C14	−0.9 (2)	C28—C23—C24—C25	0.1 (3)
C9—C8—C13—C12	0.06 (19)	O1—C23—C24—C25	−179.92 (15)
C7—C8—C13—C12	179.46 (13)	O1—C23—C28—C27	179.73 (13)
C13—C8—C9—C10	−0.7 (2)	C23—C24—C25—C26	0.0 (3)
C7—C8—C9—C10	179.95 (16)	C24—C25—C26—C27	0.1 (3)
C9—C8—C13—C14	179.71 (13)	C25—C26—C27—C28	−0.3 (3)
C8—C9—C10—C11	0.7 (3)	C26—C27—C28—C16	179.23 (14)
C9—C10—C11—C12	−0.1 (3)	C26—C27—C28—C23	0.4 (2)
C10—C11—C12—C13	−0.5 (2)	N1—C30—C31—C32	174.71 (14)
C11—C12—C13—C8	0.5 (2)	N1—C30—C35—C34	−173.82 (13)
C11—C12—C13—C14	−179.15 (14)	C35—C30—C31—C32	−1.5 (2)
C12—C13—C14—C15	−10.64 (18)	N1—C30—C35—O3	7.05 (19)
C8—C13—C14—C15	169.73 (11)	C31—C30—C35—C34	2.4 (2)
C12—C13—C14—C1	174.82 (12)	C31—C30—C35—O3	−176.78 (13)
C8—C13—C14—C1	−4.80 (18)	C30—C31—C32—C33	−0.4 (2)
C1—C14—C15—C16	−96.93 (16)	C31—C32—C33—C34	1.4 (3)
C13—C14—C15—N1	−164.56 (11)	C32—C33—C34—C35	−0.5 (3)
C13—C14—C15—C16	88.90 (14)	C33—C34—C35—C30	−1.4 (2)
C1—C14—C15—N1	9.61 (19)	C33—C34—C35—O3	177.71 (15)
N1—C15—C16—C17	−120.38 (12)		

Symmetry codes: (i) *x*, −*y*+3/2, *z*−1/2; (ii) −*x*, −*y*+1, −*z*; (iii) −*x*+1, −*y*+1, −*z*+1; (iv) −*x*, −*y*+1, −*z*+1; (v) *x*, −*y*+3/2, *z*+1/2; (vi) −*x*+1, *y*−1/2, −*z*+1/2; (vii) −*x*+1, −*y*+1, −*z*; (viii) *x*−1, *y*, *z*; (ix) −*x*+1, *y*+1/2, −*z*+1/2; (x) −*x*, *y*+1/2, −*z*+1/2; (xi) *x*+1, *y*, *z*; (xii) *x*−1, −*y*+3/2, *z*−1/2; (xiii) −*x*, *y*−1/2, −*z*+1/2; (xiv) *x*+1, −*y*+3/2, *z*+1/2.

### Hydrogen-bond geometry (Å, °)

**Table:**

*D*—H···*A*	*D*—H	H···*A*	*D*···*A*	*D*—H···*A*
C2—H2···N1	0.93	2.32	2.9236 (19)	122
C31—H31···O2	0.93	2.55	3.140 (2)	122
C5—H5···Cg1^i^	0.93	2.92	3.597 (2)	130
C26—H26···Cg2^v^	0.93	2.88	3.7180 (19)	151

Symmetry codes: (i) *x*, −*y*+3/2, *z*−1/2; (v) *x*, −*y*+3/2, *z*+1/2.
